# Systematic Investigation on Supported Gold Catalysts Prepared by Fluorine-Free Basic Etching Ti_3_AlC_2_ in Selective Oxidation of Aromatic Alcohols to Aldehydes

**DOI:** 10.3390/ma16083139

**Published:** 2023-04-16

**Authors:** Hangwei Jiang, Xiya Chen, Danlan Cui, Kun Lu, Xiao Kong, Xingguang Zhang

**Affiliations:** School of Materials and Chemistry, University of Shanghai for Science and Technology, 516 Jungong Road, Shanghai 200093, China

**Keywords:** Ti_3_AlC_2_ MAX, gold catalysts, novel reduction method, oxidation of aromatic alcohols

## Abstract

Conventional methods to prepare supported metal catalysts are chemical reduction and wet impregnation. This study developed and systematically investigated a novel reduction method based on simultaneous Ti_3_AlC_2_ fluorine-free etching and metal deposition to prepare gold catalysts. The new series of Au_pre_/Ti_3_Al_x_C_2_Ty catalysts were characterized by XRD, XPS, TEM, and SEM and were tested in the selective oxidation of representative aromatic alcohols to aldehydes. The catalytic results demonstrate the effectiveness of the preparation method and better catalytic performances of Au_pre_/Ti_3_Al_x_C_2_T_y_, compared with those of catalysts prepared by traditional methods. Moreover, this work presents a comprehensive study on the influence of calcination in air, H_2_, and Ar, and we found that the catalyst of Au_pre_/Ti_3_Al_x_C_2_T_y_-Air600 obtained by calcination in air at 600 °C performed the best, owing to the synergistic effect between tiny surface TiO_2_ species and Au NPs. The tests of reusability and hot filtration confirmed the catalyst stability.

## 1. Introduction

A heterogeneous catalyst is a material that alters the path of a chemical reaction without itself being expended, and this unique characteristic allows a small amount of catalyst mass to convert a large quantity of reactant without undergoing a stoichiometric reaction pathway [1,2,3]. The quest for new catalytic materials or novel preparation methods underpins the development of catalysis science, technology, and engineering applications [4,5].

In the recent decade, a new family of 2D materials of MAX phases have attracted researchers’ interest [6], and these materials possess typical ternary metal carbides, nitrides, or carbonitrides with layered hexagonal crystal structures. As the name (MAX) indicates, their general chemical formula is denoted as M_n+1_AX_n_, where M is an early transition metal, A is an element (mostly from Groups 13 and 14, e.g., Al or Si), X is carbon and/or nitrogen, and n = 1, 2, or 3. They have a combination of covalent and metallic properties, exhibit a unique combination of ceramic and metallic properties, and conduct heat and electricity like metals, yet they are elastically stiff, strong, and brittle, and some are heat-tolerant like ceramics. Numerous studies have been published on the electrical, thermal, and mechanical properties of the MAX phases and their derived MXenes (graphene-like nanosheets) with functional surface species after treatment with strong acidic exfoliation [7,8]. However, the use of MAX phases as catalysts had been ignored until 2017, when Ng and co-workers showed the potential of a typical MAX of Ti_3_AlC_2_ in selective oxidation reactions (oxidative dehydrogenation of n-butane [9]). Recently, Trandafir and co-workers reported that the MAX phase powders of Ti_3_SiC_2_, Ti_2_AlC, or Ti_3_AlC_2_ can chemoselectively hydrogenate 4-nitrostyrene to 4-aminostyrene with 100% selectivity at around a 3–4% conversion; while loading Ti_3_SiC_2_ with 0.0005wt% of Pd and increasing the conversion to 100%, while maintaining the 4-aminostyrene selectivity at >90% [10]. Slot and co-workers reported that Ru supported on alkali-exfoliated Ti_3_(Al_0.8_Sn_0.2_) C_2_ MAX phase and improved reactivity, possibly related to metal–support electronic interaction [11]. These studies are pioneering work in applying MAX phases in catalysis, and the catalytic applications are attracting increasing attention [12,13].

In addition to the rareness of MAX phase applications in catalysis, the popular transformation of MAX phases requires strong acid, such as concentrated hydrofluoric acid (50 wt% HF aqueous solution [14]) to selectively remove A layers (such as Al in Ti_3_AlC_2_), giving rise to environmental harm and decreasing the material performance because the high-concentration fluorine ions are inert surface terminals. New fluorine-free methods for the removal of A layers are urgently needed, and recent years have seen phenomenal progress in utilizing basic solutions (e.g., NaOH aqueous solution) to etch MAX phases [15,16]. Our previous study also found that when Ti_3_AlC_2_ was etched by NaOH aqueous solution under hydrothermal reactions (150 °C–250 °C), the solid products could be nanosheets, nanofibers, nanobelts, or nanoparticles (NPs), relying on the conditions (e.g., alkaline concentration, reaction temperature, and time) [17]. Moreover, peer studies reported that the following reactions probably occurred when Ti_3_AlC_2_ was etched in the NaOH solution [15,18]: Ti_3_AlC_2_ + (1 – x + y) NaOH + (1 – x) H_2_O = Ti_3_Al_x_C_2_(ONa)_y_ + (1 – x) NaAlO_2_ + 0.5(3 – 3x + y) H_2_ and Ti_3_AlC_2_ + (1 – x) NaOH + (1 – x + z) H_2_O = Ti_3_Al_x_C_2_(OH)_z_ + (1 – x) NaAlO_2_ + 0.5(3 – 3x + z) H_2_ (0 ≤ x < 1, 1 ≤ y or z < 2). Inspired by the above studies, we deduced that the as-produced H_2_ should be able to simultaneously reduce metal cationic precursors to fabricate supported metal on the Ti_3_AlC_2_-derived nanomaterials (denoted as Ti_3_Al_x_C_2_T_y_, T = ONa/OH or =O, 0 < x < 1, 1 < y < 2). Fortunately, this hypothesis was verified in our recent study, which demonstrated that a series of Pt/Ti_3_Al_x_C_2_T_y_ catalysts were successfully achieved and outperformed their counterparts prepared by conventional wet impregnation, deposition–precipitation, or chemical reduction in the aqueous-phase selective hydrogenation of furfural to furfuryl alcohol [19].

Supported metal NPs are important categories of heterogeneous catalysts and are widely applied in the chemical industry and in environmental remediation [1,4,20,21,22]. Supported gold catalysts, after pioneering work by Hutchings [23], Haruta [24], and Goodman [25], have been attracting tremendous attention in both academic and engineering research [26]. Reported methods to prepare supported metal catalysts are usually by chemical reduction and wet impregnation. In this study, we employed chloroauric acid (HAuCl_4_) as the metal precursor and succeeded in preparing supported gold catalysts on Ti_3_Al_x_C_2_T_y_, using simultaneous Ti_3_AlC_2_ etching and Au deposition as reduced by H_2_ released under hydrothermal conditions. The catalytic performances of the as-obtained gold catalysts were examined in the selective oxidation of aromatic alcohols into their corresponding aldehydes, which are widely used in the fields of fragrances, additives, and pharmaceuticals [27,28]. Moreover, the new catalysts were also systematically investigated as compared with their counterparts prepared by traditional methods, and they were also calcined in air, Ar, and H_2_ at different temperatures (200, 400, and 600 °C) to explore the influential factors on catalytic performances. The calcined catalysts in air-formed partially oxidized surface species (e.g., TiO_2_) exhibited synergetic interactions with Au NPs and boosted the catalytic activity [29], whereas calcined catalysts in H_2_ and Ar declined the activity because of serious aggregation of Au NPs. The findings of this work should help peer researchers to rationally design new supported metal or alloy catalysts [30,31] and other MAX phase-derived supports [8,12].

## 2. Materials and Methods

### 2.1. Materials

Ti_3_AlC_2_ powder (98% purity, 400 mesh) was purchased from Liaoning 11 Sci-Tech Co., LTD. (Changchun, China). Chloroauric acid (HAuCl_4_) was purchased from Macklin (Shanghai, China). Sodium hydroxide (≥96%, NaOH) and urea (99%) were purchased from Sinopharm Chemical Reagent Company Co., Ltd. (Shanghai, China). Benzyl alcohol (99%), benzaldehyde (99%), benzoic acid (99%), toluene (99.8%), and sodium borohydride (NaBH_4_, 98%) were purchased from Shanghai Titan Technology Co., LTD. (Shanghai, China). Cinnamic alcohol (98%) was purchased from Shanghai Xushuo biotechnology Co., Ltd. (Shanghai, China). 4-Methylbenzyl alcohol (98%), 4-methoxylbenzyl alcohol (98%), and poly (diallyldimethylammonium chloride) solution (PDDA, 20 wt% in water) were purchased from Aladdin Industrial Inc., Shanghai, China.

### 2.2. Preparation of Ti_3_Al_x_C_2_T_y_ Supports

The supports of Ti_3_Al_x_C_2_T_y_ (T refers to -OH/Na or =O, 0 < *x* < 1, 1 < *y* < 2) were prepared according to the optimized procedure reported in our previous study [17]. Typically, 1 g of Ti_3_AlC_2_ powder was added into 15 mL of 10 M NaOH aqueous solution and stirred at room temperature for 1 h. Then, the suspension was transferred into a 100 mL autoclave with a PTFE container inside to undergo heating at 180 °C for 48 h. The solid products were washed with deionized water to pH = 7–9 and then were dried in an oven at 80 °C overnight. Before use, the dried products were ground in a mortar into powder to obtain Ti_3_Al_x_C_2_T_y_.

### 2.3. Preparation of Au_pre_/Ti_3_Al_x_C_2_T_y_ Catalysts

The details of the novel reduction method were the following: 1 g of Ti_3_AlC_2_ powder was mixed with 15 mL of 10 mol·L^−1^ NaOH aqueous solution. The suspension was continuously stirred for 1 h at room temperature. Then, the desired amount of HAuCl_4_ aqueous solution with preset concentration was added with continuous stirring for 1 h. The solution was subsequently transferred into a 100 mL autoclave with a PTFE container inside. The autoclave was then maintained at 180 °C for 48 h under static conditions in an oven. The final solid products were collected by filtration and washed thoroughly with deionized water until the pH = 7–9. The nominal loading of gold was 0.25wt%, 0.5wt%, 1wt%, 1.5wt%, and 2wt%, which was obtained by changing the amount of HAuCl_4_ aqueous solution. The final catalysts were correspondingly denoted as 0.25wt%-, 0.5wt%-, 1wt%-, 1.5wt%-, and 2wt%Au_pre_/Ti_3_Al_x_C_2_T_y_. Based on catalysts screening results, the optimistic gold loading was 1wt%, and the following study focused on this typical loading of gold.

### 2.4. Thermal Treatment Procedures

Typically, 1 g of catalysts or supports was put in a ceramic boat in a tube furnace, and then the system was purged by air for 20 min at a gas flow rate of 40 mL/min. The furnace was heated at a rate of 5 °C/min to the target temperature of 200 °C, 400 °C, and 600 °C and then maintained for 2 h, followed by cooling naturally to room temperature. The series of catalysts obtained were denoted as Au_pre_/Ti_3_Al_x_C_2_T_y_-Air200, Au_pre_/Ti_3_Al_x_C_2_T_y_-Air400, and Au_pre_/Ti_3_Al_x_C_2_T_y_-Air600. Similarly, the catalysts that were treated in H_2_ were denoted as Au_pre_/Ti_3_Al_x_C_2_T_y_-H200, Au_pre_/Ti_3_Al_x_C_2_T_y_-H400, and Au_pre_/Ti_3_Al_x_C_2_T_y_-H600, and those treated in Ar were denoted as Au_pre_/Ti_3_Al_x_C_2_T_y_-Ar200, Au_pre_/Ti_3_Al_x_C_2_T_y_-Ar400, Au_pre_/Ti_3_Al_x_C_2_T_y_-Ar600.

### 2.5. Preparation of Control Catalysts by the Chemical Reduction (cr) Method

The control catalyst of Au_cr_/Ti_3_Al_x_C_2_T_y_ was prepared by the chemical reduction method according to our previous study [32]. Typically, in a glass beaker with a magnetic stirring bar, 2.5 g of Ti_3_Al_x_C_2_T_y_ supports was dispersed into a certain amount of aqueous solution of HAuCl_4_ (1.0 g of HAuCl_4_ dissolved in 100 mL of water) to achieve a nominal loading of Au (e.g., 1wt%). Subsequently, 20 mL of 0.53 mol/L lysine aqueous solution was added dropwise to the mixture under magnetic stirring, and after that, the stirring was prolonged for 30 min. To this suspension, 10 mL of 0.35 mol/L NaBH_4_ aqueous solution was added dropwise, followed by 10 mL of 0.3 mol/L HCl aqueous solution to achieve pH = 9.5. The mixture was stirred for 1 h and aged for 24 h. Then, the solid was separated and washed firstly with sufficient deionized water three times to remove the chlorine ions; the residual solution was tested by 0.1 mol/L AgNO_3_ aqueous solution, and no AgCl precipitate was observed. The solids were collected and dried at 60 °C for 16 h.

### 2.6. Preparation of Control Catalysts by the Colloidal Deposition (col) Method

The gold colloidal suspension was prepared firstly, in which 10 mL of KOH (0.1 mol/L) was added to 10 mL HAuCl_4_ and stirred for 10 min. Then, 0.3 g of PDDA solution was completely diluted in 10 mL of water, and then it was added dropwise to the above solution and stirred for 10 min. After that, 10 mL of NaBH_4_ (0.5 mol/L) was added dropwise and then stirred for 1 h, and the colloidal suspension was aged for 24 h. In the synthesis of catalysts, the required amount of gold colloidal suspension was used directly. The other procedures are given in the section of “Preparation of Au_pre_/Ti_3_Al_x_C_2_T_y_ catalysts”, and the final catalysts were denoted as Au_col_/Ti_3_Al_x_C_2_T_y._

### 2.7. Preparation of Control Catalysts by the Wet Impregnation (wi) Method

Typically, 0.2 g of supports was dispersed in 10 mL of water. The desired amount of chloroauric acid (HAuCl_4_) was dissolved into 20 mL of water according to the nominal loading, followed by adjusting pH = 10. The above support suspension was added to the HAuCl_4_ solution under stirring for 18 h. Finally, the solid products were washed and dried at 60 °C for 16 h. The obtained solid powders were named as Au_wi_/Ti_3_Al_x_C_2_T_y_.

### 2.8. Characterizations

The crystal structures were analyzed by X-ray diffraction (XRD) using Rigaku Smartlab with Cu Kα radiation (λ = 0.1542 nm) at a scan rate of 5°/min from 5 to 80 (2θ) at a voltage of 40 Kv. The morphologies of the samples were examined by scanning electron microscopy (SEM) utilizing a JSM-7600F (JEOL Ltd., Tokyo, Japan) with an operating voltage of 30 Kv. The morphologies of the target materials and the diameters of Au NPs were also examined by transmission electron microscopy (TEM) utilizing a JEOL JEM-2100 apparatus with an operating voltage of 200 Kv. XPS (X-ray photoelectron spectroscopy) was performed on a Kratos Axis Hsi X-ray photoelectron spectrometer fitted with a charge neutralizer and magnetic focusing lens, employing Al Kα monochromatic radiation (1486.7 Ev). CasaXPS version 2.3.16 was used for spectra fitting. Binding energies were corrected to the C 1s peak at 284.8 Ev, and surface atomic compositions were calculated via correction for the appropriate instrument response factors. Au4f spectra were fitted using a Doniach Sunjic modified Gaussian–Lorentzian asymmetric line shape. The real loading gold content was detected by the Inductive Coupled Plasma (ICP) on NeXion 300X (PE Ltd., Waltham, MA, USA) instrument, and aqua regia was used for digestion under ultrasonic condition for 4 h.

### 2.9. Catalytic Reactions

The catalyst was evaluated by aerobic oxidation of benzyl alcohol in the dark, and the experimental setup was on the basis our previous study [27,32]. The reaction was conducted in a 100 mL round-bottomed Pyrex glass flask with a sealed rubber spigot and a magnetic stirrer. The reaction temperature was controlled by an oil bath to maintain 60 °C. Typically, 4 mmol of benzyl alcohol was dissolved into 20 mL of toluene as solvent, and 0.4 mmol of NaOH (molar ratio 0.4/4 = 10%) and 200 mg of the catalysts were added into the mixture (the influence of mass transfer and adsorption under these conditions were ruled out according to our previous study [27,32]). The suspension of catalysts and reaction solution were stirred for 2 h to rule out the adsorption effect; then, a control sample was collected. For sampling, 1 mL of aliquots was collected and filtered through a Millipore nylon filter (pore size 0.22 μm) to remove the catalyst particulates and to saturate the filter; then, another 1 mL of reaction solution was collected and filtered using the same sampler. Subsequently, the suspension was purged with O_2_ for three minutes to replace air and then sealed, and the reactor was submerged into the oil bath, which had been preheated to 60 °C. After a certain time period (1 h, 2 h, 4 h, and 6 h), 1 mL of reaction solution was collected. To test other reactants, 4 mmol of another kind of aromatic alcohol was also tested under identical conditions.

The detection and quantification of reactants and products in the solution were conducted on a Gas Chromatograph (GC 9720P, produced by Zhejiang FULI, Taizhou, China) equipped with the HP-5 column (Agilent J&W GC Columns, HP-5, length 30 m, diameter 0.320 mm, and film thickness 0.25 microns). The quantification method was on the basis of our previous work [33], and to illustrate, analyses of each sample were performed in triplicate, with a peak area reproducibility of ±2% for standards, such as benzyl alcohol and benzaldehyde, and ±10% for those samples if the concentration of product was very low. Response factors for each aromatic alcohol and their corresponding aldehyde were determined from respective multipoint calibration curves. The conversion of aromatic alcohols and product selectivity were calculated according to the following equations:

(1) $$\mathrm{Conversion}\;(\%)=\frac{{\mathrm{moles}\;\mathrm{of}\;\mathrm{reactant}}_{t=0}{\;-\;\mathrm{moles}\;\mathrm{of}\;\mathrm{reactant}}_{t=6}\;}{{\mathrm{moles}\;\mathrm{of}\;\mathrm{reactant}}_{t=0}}\times 100$$

(2) $$\mathrm{Selectivity}\;(\%)=\frac{{\mathrm{moles}\;\mathrm{of}\;\mathrm{product}}_{t=6}\;}{{\mathrm{moles}\;\mathrm{of}\;\mathrm{reactant}}_{t=0}{-\mathrm{moles}\;\mathrm{of}\;\mathrm{reactant}}_{t=6}}\times 100$$

The terminal reaction rate (mmol_reactant_·g_Au_^−1^·h^−1^) was calculated according to the following equation based on the real loading of gold content detected by ICP:

(3) $$\mathrm{Reaction}\;\mathrm{rate}=\frac{\mathrm{Number}\;(\mathrm{mmol})\;\mathrm{of}\;\mathrm{benzyl}\;\mathrm{alcohol}\;\mathrm{converted}}{\mathrm{Gram}\;\mathrm{of}\;\mathrm{gold}\;\mathrm{loading}\;\times \;\mathrm{Reaction}\;\mathrm{time}\;(h)}$$

## 3. Results and Discussion

### 3.1. Crystal Structures of Catalysts by XRD

**Figure 1:**
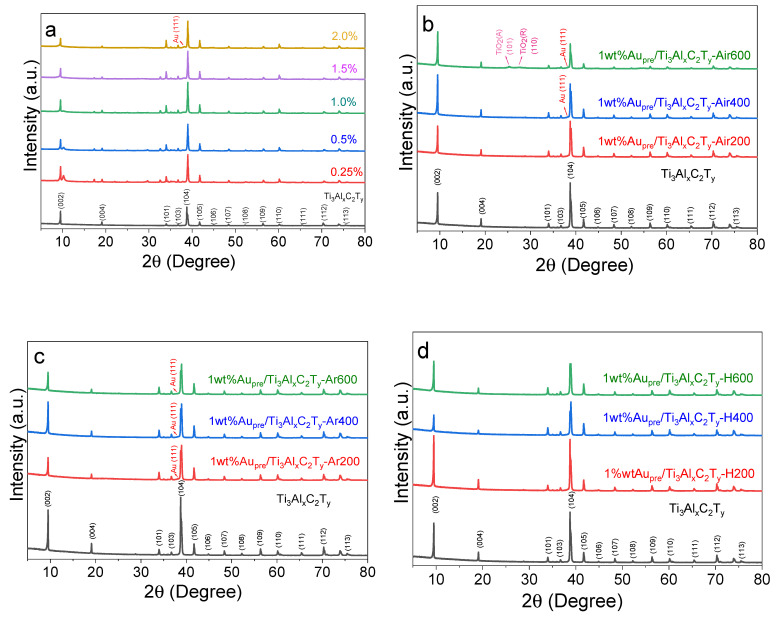
XRD patterns of four series of catalysts (**a**) Au_pre_/Ti_3_Al_x_C_2_T_y_ with different nominal gold loadings (wt%), (**b**) series of 1wt%Au_pre_/Ti_3_Al_x_C_2_T_y_-Air catalysts calcined in air, (**c**) series of 1wt%Au_pre_/Ti_3_Al_x_C_2_T_y_-Ar calcined in Ar, and (**d**) series of 1wt%Au_pre_/Ti_3_Al_x_C_2_T_y_-H catalysts calcined in H_2_.

The series of Aupre/Ti_3_Al_x_C_2_T_y_ catalysts with different gold loadings were originally prepared by the novel reduction method based on simultaneous Ti_3_AlC_2_ basic etching. XRD characterizations were performed to examine their crystal structures. As shown in Figure 1a, altering the content of gold loading did not change the crystal structure of Ti_3_Al_x_C_2_T_y_; moreover, the alkali etching under the current hydrothermal conditions could maintain the primary crystal structures of the parent MAX phase of Ti_3_AlC_2_ as reported in the literature [34,35], and the detailed XRD patterns of Ti_3_AlC_2_ (Figure S1a) and enlarged XRD patterns of the series of Au_pre_/Ti_3_Al_x_C_2_T_y_ catalysts are displayed in Figure S1b–f.

Figure 1b displays the XRD patterns of the series of 1wt%Au/Ti_3_Al_x_C_2_T_y_-Air catalysts calcined in air and compared with the XRD pattern of Ti_3_Al_x_C_2_T_y_ support. The relatively low-temperature calcination at 200 °C and 400 °C showed no identifiable change in the diffraction peaks compared with those of Ti_3_Al_x_C_2_T_y_. After calcination at 600 °C, the primary crystal structures of Ti_3_Al_x_C_2_T_y_ still dominated, whereas tiny diffraction peaks of anatase TiO_2_ (101) and rutile TiO_2_ (110) could be identified, and they likely formed on the surface of Ti_3_Al_x_C_2_T_y_. To confirm the formation of surface TiO_2_ species, the HR-TEM image of 1wt%Au/Ti_3_Al_x_C_2_T_y_-Air600 was conducted as shown in Figure 3e. In addition, Ti_3_Al_x_C_2_T_y_ was also calcined in air at 200 °C, 400 °C, and 600 °C with other conditions being identical, and the XRD patterns are compared in Figure S2. Increasing the calcination temperature did not alter the main crystal structures of Ti_3_Al_x_C_2_T_y_; however, the peak intensity declined slightly, and small peaks of anatase TiO_2_(101) and rutile TiO_2_(110) could also be detected.

As shown in Figure 1c, the XRD patterns of the series of 1wt%Au_pre_/Ti_3_Al_x_C_2_T_y_ catalysts calcined in Ar were basically consistent with those catalysts calcined in H_2_ (Figure 1d). Interestingly, detailed analyses showed that the diffraction characteristic peaks of Au NPs on 1wt%Au_pre_/Ti_3_Al_x_C_2_T_y_-H were less observable compared with those on 1wt%Au_pre_/Ti_3_Al_x_C_2_T_y_-Ar, possibly because of the generation of strong metal–support interaction (SMSI) that resulted in the encapsulation of Au NPs in H_2_ calcination [36].

### 3.2. Oxidation States of Gold by XPS Analyses

**Figure 2:**
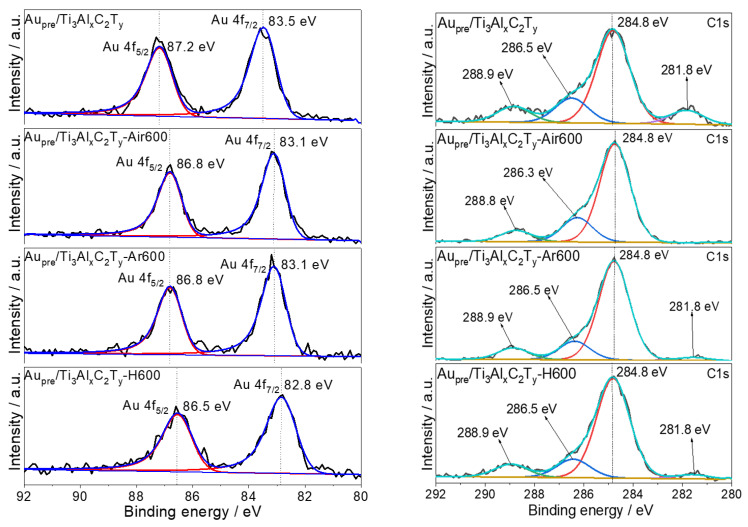

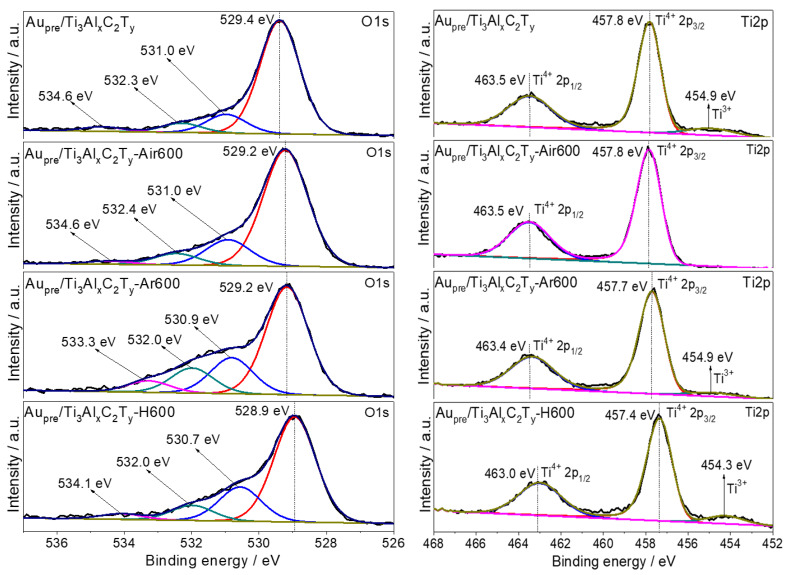
High-resolution XPS analyses Au4f, C1s, O1s, and Ti2p over catalysts of Au_pre_/Ti_3_Al_x_C_2_T_y_, 1wt%Au_pre_/Ti_3_Al_x_C_2_T_y_-Air600, 1wt%Au_pre_/Ti_3_Al_x_C_2_T_y_-Ar600, and 1wt%Au_pre_/Ti_3_Al_x_C_2_T_y_-H600.

One of the main aims of this study is to prove that metallic Au deposits on the series of Au_pre_/Ti_3_Al_x_C_2_T_y_ catalysts prepared by the novel reduction method based on simultaneous basic etching. Hence, it is imperative to examine the oxidation states of Au4f by detailed analyses of high-resolution XPS. As expected, metallic Au was achieved without using any extra reductive agents, and the high-resolution XPS spectra of Au 4f are given in Figure 2, showing the binding energies of 83.5 eV (Au 4f_7/2_) and 87.2 eV (Au 4f_5/2_) [37,38].

To further confirm the effectiveness of this novel reduction method, a control catalyst was prepared by the traditional chemical reduction method using the strong reductive agent of NaBH_4_ and the premade Ti_3_Al_x_C_2_T_y_ as supports (denoted as Au_cr_/Ti_3_Al_x_C_2_T_y_). The high-resolution XPS spectra of Au4f (Figure S3) proved that the binding energies were 83.7 eV (Au 4f_7/2_) and 87.4 eV (Au 4f_5/2_) on the control catalyst, also corresponding to metallic gold. For both Au_pre_/Ti_3_Al_x_C_2_T_y_ and Au_cr_/Ti_3_Al_x_C_2_T_y_, negligibly positively/negatively charged gold species were identified (please see Table S1 for more summarized information on the binding energies of metallic and positively charged and negatively charged gold species on various supports). These results demonstrate that the novel reduction strategy was essentially as efficient as the chemical reduction method, and it is more cost-effective as H_2_ was released from the transformation of raw Ti_3_AlC_2_.

After calcination in air, Ar, or H_2_ at 600 °C, the binding energies of Au4f_7/2_ shifted to a lower binding energy of 83.1 eV for both 1wt%Au/Ti_3_Al_x_C_2_T_y_-Air600 and 1wt%Au/Ti_3_Al_x_C_2_T_y_-Ar600 and 82.8 eV over 1wt%Au/Ti_3_Al_x_C_2_T_y_-H600. This slight shift was probably caused by the electron transfer through the support–metal (gold) interface, owing to the strong electronegativity of gold as reported in the literature [38,39], thereby resulting in partially negatively charged gold species (more information is provided in Table S1).

As to other key elements of C1s, O1s, and Ti2p, the high-resolution XPS spectra were also analyzed (Figure 2), offering critical information on the surface species of catalysts. The C1s spectra of all catalysts showed the existence of C–C centered at 284.8 eV, C–O cantered at 286.5 eV, and C=O/O–C=O centered at 288.9 eV [40,41]. Moreover, the peak centered at 281.8 eV signified the existence of carbide species over 1wt%Au/Ti_3_Al_x_C_2_T_y_ [42]. This peak declined over samples treated in Ar and H_2_ at 600 °C and disappeared after calcination in air at 600 °C, indicating that the surface carbide species of 1wt%Au/Ti_3_Al_x_C_2_T_y_ transformed into TiO_2_ species.

The high-resolution XPS spectra of O1s over the four catalysts have four characteristic peaks centred around 529.2 eV, 531.0 eV, 532.0 eV, and 534.1 eV, which could be assigned to the lattice oxygen (e.g., Ti-O), bridging oxygen (e.g., Ti=O), oxygen vacancies, top-site oxygen (e.g., -OH/Na or =O) [43], or surface-adsorbed oxygen species [44].

As to the Ti2p, two major spin-orbital peaks of Ti^4+^ were observed. The Ti2p_3/2_ peak centered at 457.8 eV (Ti^4+^) over four catalysts [45]; however, they varied as to peaks of Ti^3+^ located at 454.9 eV [46], because different calcination atmospheres affected the content of Ti^3+^ and it nearly disappeared after calcination in air (possible due to the reaction: Ti^3+^ + 1/2O_2_-e^−^ → Ti^4+^-O). When calcined in Ar or H_2_, the Ti^3+^ sites possibly lost electrons to form negatively charged gold species (Ti^3+^-e^−^ + Au → Ti^4+^ + Au^δ−^); hence, this electron transfer from carrier to Au could explain the shift of Au4f to lower-binding energies after calcination treatment (Figure 2) [47].

### 3.3. Size and Morphological Analyses

**Figure 3:**
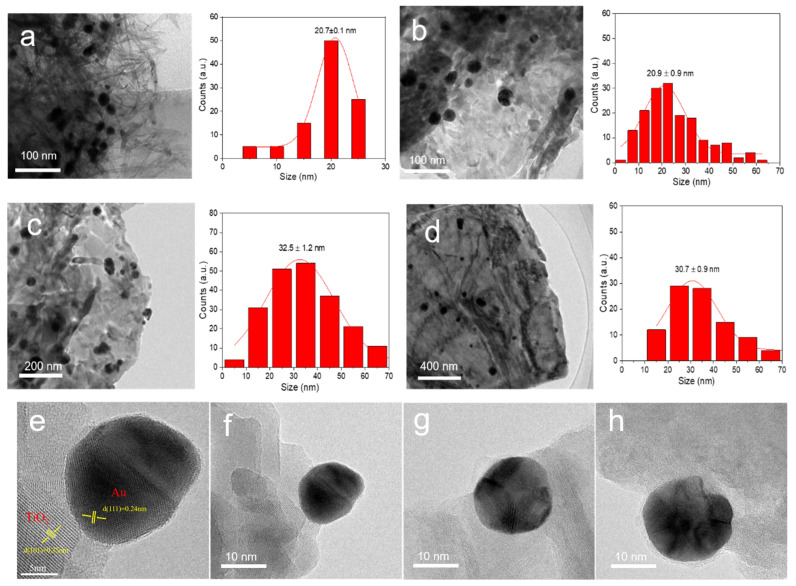
TEM images and size-distribution histograms of Au NPs of (**a**) 1wt%Au/Ti_3_Al_x_C_2_T_y_, (**b**) 1wt%Au/Ti_3_Al_x_C_2_T_y_-Air600, (**c**) 1wt%Au/Ti_3_Al_x_C_2_T_y_-Ar600, and (**d**) 1wt%Au/Ti_3_Al_x_C_2_T_y_-H600. RTEM image of (**e**–**f**) 1wt%Au/Ti_3_Al_x_C_2_T_y_-Air600, (**g**) 1wt%Au/Ti_3_Al_x_C_2_T_y_-Ar600, and (**h**) 1wt%Au/Ti_3_Al_x_C_2_T_y_-H600.

The particle size of Au NPs and the morphology of catalysts were examined by TEM and SEM. As shown in Figure 3, the mean size of Au NPs on 1wt%Au_pre_/Ti_3_Al_x_C_2_T_y_ and 1wt%Au_pre_/Ti_3_Al_x_C_2_T_y_-Air600 were 20.7 nm and 20.9 nm, respectively, revealing that calcination in air did not give rise to the aggregation of Au NPs. Interestingly, the calcination in air at different temperatures did not change the mean particle size of Au NPs (Figure S4). The HR-TEM image in Figure 3e shows that calcination in air at 600 °C gave rise to the formation of tiny TiO_2_ on the surface, and two sets of lattice stripes were observed with a lattice space of 0.35 nm and 0.24 nm [S1], which belong to the (101) faces of TiO_2_ and the (111) faces of Au, respectively [29,48]. However, the mean size of Au NPs on 1wt%Au_pre_/Ti_3_Al_x_C_2_T_y_-Ar600 was 32.5 nm, and it was 30.7 nm on 1wt%Au_pre_/Ti_3_Al_x_C_2_T_y_-H600, demonstrating obvious aggregation of Au NPs and a possible strong interaction between Au NPs and supports; in particular, an amorphous overlayer could be observed on Au NPs over 1wt%Au_pre_/Ti_3_Al_x_C_2_T_y_-H600 (HR-TEM images are given in Figure 3f–h) [36,49]. The aforementioned results show that the mean particle size of Au NPs prepared by the novel reduction method was relatively larger than those (generally < 10 nm) prepared by the traditional chemical reduction method as reported in our previous studies [27,32].

**Figure 4:**
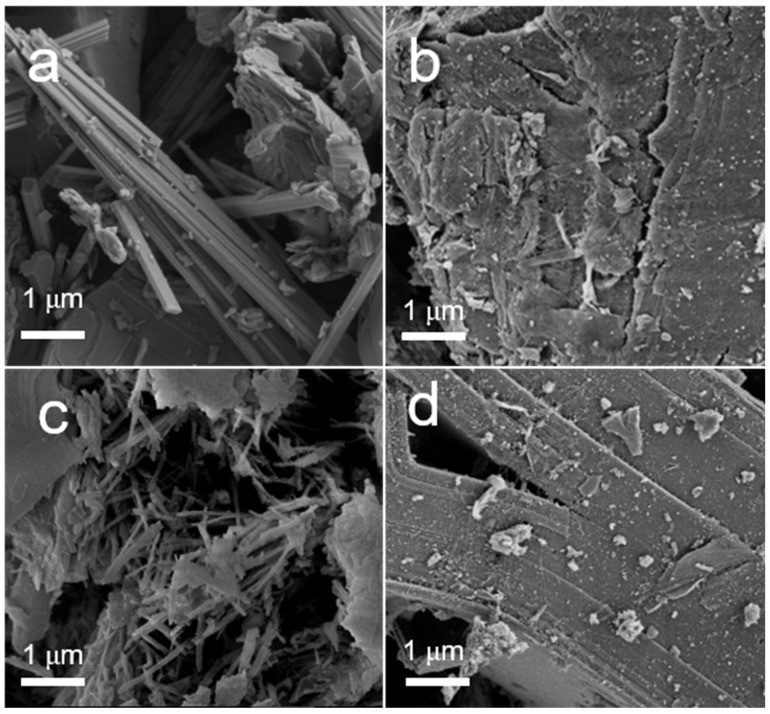
SEM images of supports and typical catalysts: (**a**) 1wt%Au_pre_/Ti_3_Al_x_C_2_T_y_, (**b**) 1wt%Au_pre_/Ti_3_Al_x_C_2_T_y_-Air600, (**c**) 1wt%Au_pre_/Ti_3_Al_x_C_2_T_y_-Ar600, and (**d**) 1wt%Au_pre_/Ti_3_Al_x_C_2_T_y_-H600.

Figure 4 displays the SEM images of the four representative catalysts: 1wt%Au_pre_/Ti_3_Al_x_C_2_T_y_, 1wt%Au_pre_/Ti_3_Al_x_C_2_T_y_-Air600, 1wt%Au_pre_/Ti_3_Al_x_C_2_T_y_-Ar600, 1wt%Au_pre_/Ti_3_Al_x_C_2_T_y_-H600. As shown in Figure 4a and Figure S5a, the newly synthesized 1wt%Au_pre_/Ti_3_Al_x_C_2_T_y_ showed a fibre-like structure, not a dense layered structure, such as Ti_3_AlC_2_. After calcination in air at 600 °C, noticeably tiny TiO_2_ nanocrystals formed on the surface, as shown in Figure 4b and Figure S5b, and the nanocrystals were uniform in size and morphology (as mentioned above, the existence of TiO_2_ nanocrystals was also detected by XRD patterns and HR-TEM analysis). Figure 4c and Figure S5c show that the calcined sample 1wt%Au_pre_/Ti_3_Al_x_C_2_T_y_-Ar600 still retained the fibre-like morphology, whereas the H_2_-treated sample 1wt%Au_pre_/Ti_3_Al_x_C_2_T_y_-H600 had denser layered structures, as shown in Figure 4d and Figure S5d.

### 3.4. Catalytic Tests and Catalyst Screenings

To evaluate the catalytic performances of the aforementioned new catalysts and the effect of influential factors, the selective oxidation of aromatic alcohol to their corresponding aldehyde was selected as model reactions [50]. A series of preliminary experiments as to the effect of reaction parameters on the catalytic activity were first carried out on the basis of our previous study [27], and the detailed optimization work of this study is provided in Figures S6–S9, Section 7 in Supplementary Information. As shown in Table 1, the control experiment without catalysts and reactions using Ti_3_AlC_2_ and Ti_3_Al_x_C_2_T_y_ as catalysts exhibited negligible conversions. As for the catalysts with different mass loadings of gold, both the conversion and selectivity gradually increased as the function of Au loadings increased. When it came to the reaction rate, the catalyst of 1wt%Au_pre_/Ti_3_Al_x_C_2_T_y_ showed the highest value (110.4 mmol·g_Au_^−1^·h^−1^); therefore, the 1wt%Au_pre_/Ti_3_Al_x_C_2_T_y_ catalyst was selected as a model catalyst for further study on thermal treatment, and the gold loading of 1wt% was employed as a typical loading.

By comparing the catalytic performances of catalysts calcined under varying temperatures (200 °C, 400 °C, or 600 °C) or atmospheres (H_2_, Ar, or air), we found that the catalytic activity of the catalysts calcined in air significantly improved, whereas the catalytic activity of the catalyst calcined by Ar and H_2_ noticeably declined. We deduced that air calcination altered the surface of supports into TiO_2_-like species as revealed by XRD diffractions (Figure 1b). It has been well established that TiO_2_ can catalyse the conversion of benzyl alcohol into benzaldehyde [51]; moreover, researchers reported that Ti_3_C_2_ MXenes calcined to give TiO_2_/Ti_3_C_2_ hybrids also exhibited a high conversion of benzyl alcohol to benzaldehyde [29]. On the contrary, calcination under the inert gas of Ar or the reductive gas of H_2_ led to an obvious aggregation of Au NPs or the formation of amorphous overlayers on Au NPs (Figure 3g–h) [52]. Among these catalysts, the optimized catalyst was 1wt%Au_pre_/Ti_3_Al_x_C_2_T_y_-Air600, which gave a conversion of 38.5%, and the reaction rate was 234.0 mmol g_Au_^−1^ h^−1^, almost doubling the reaction rate of the uncalcined 1wt%Au_pre_/Ti_3_Al_x_C_2_T_y_ and tripling that of 2wt%Au_pre_/Ti_3_Al_x_C_2_T_y_. Nevertheless, it is noteworthy that decent conversions or excellent selectivity were also obtained over catalysts, such as 1wt%Aupre/Ti_3_Al_x_C_2_T_y_, 1wt%Aupre/Ti_3_Al_x_C_2_T_y_-Air400, and 1wt%Aupre/Ti_3_Al_x_C_2_T_y_-Ar200. They demonstrated that the TiO_2_ species could boost conversions, but they could only be obtained at higher calcination temperatures, which compromises the economic rationale for catalyst preparation process.

Selectivity is also an important criterion for evaluating the performance of a catalyst. As shown in Table 1, when the gold loading was 0.25wt% and 0.5wt%, the selectivity for benzaldehyde was only 61.4% and 68.8%, respectively. When the gold loading reached 1wt%, the selectivity improved to 88.2%, substantially proving that the loading of Au determined the selectivity of benzaldehyde. Comparing the catalytic performances of supported gold catalysts on other supports from reported studies (Table S3), the newly developed catalysts in this study exhibited better catalytic performances for liquid-phase benzyl alcohol oxidation to benzaldehdye under similar reaction conditions.

The catalytic results in Table 1 encourage us to further investigate the efficiency of this novel reduction method by comparing with other traditional preparation methods of supported metal catalysts, such as the colloidal deposition method [53], the chemical reduction method [54], and the wet impregnation method [55] (experimental details can be found in the experimental parts). Moreover, the three catalysts were also calcined at 600 °C to see the change in catalytic performances. As shown in Table 2, the catalytic performances of the catalysts prepared by the novel reduction method outperformed the other three catalysts prepared by traditional methods. Correspondingly, after calcination in air, the Au_pre_/Ti_3_Al_x_C_2_T_y_-Air600 catalyst stilled exhibited the best performances. Hence, it is reasonable to deduce that improved performances are due to the formation of close contact between Au NPs and supports under hydrothermal transformation [5], and this will be discussed in the following section about the “synergistic effect test”.

### 3.5. Effect of Different Reactants

The general applicability of the new catalyst was evaluated using several kinds of aromatic alcohols over 1wt%Au_pre_/Ti_3_Al_x_C_2_T_y_-Air600, and the results are shown in Table 3. Except for cinnamyl alcohol, the other three representative reactants showed higher reaction rates, indicating that 1wt%Au_pre_/Ti_3_Al_x_C_2_T_y_-Air600 could be a versatile catalyst to produce a range of aromatic aldehydes. The reasons for the difference in conversion of these reactants have been investigated in many studies, involving electronic effects, steric effects, adsorptive energies, molecular polarities of reactants, and the like [27,32]. For example, for the parasubstituted reactants, the catalytic activity followed the Hammett rule: accordingly, substitution with electron-donating groups, such as -OCH_3_ and -CH_3_, exhibited higher conversions [56]. In the current stage, it is challenging to exactly quantify the individual contribution of each influential factor based on experimental results; nonetheless, these results show that 1wt%Au_pre_/Ti_3_Al_x_C_2_T_y_-Air600 is a potential candidate for applications in a large scale, because the fibrous catalysts are more facile to recycle from liquid catalytic systems for reuse compared with nanoparticulate catalysts.

### 3.6. Stability Evaluation by Tests of Reusability and Hot Filtration

**Figure 5:**
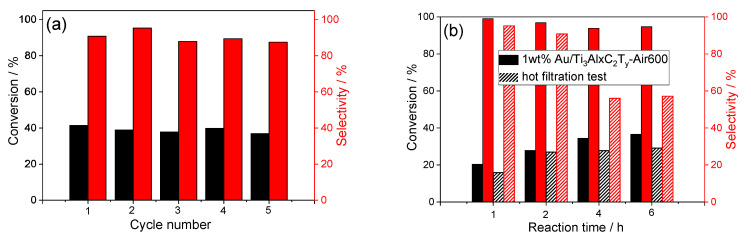
Tests of (**a**) reusability and (**b**) hot filtration to examine leaching of active species. Reaction conditions: Catalyst (200 mg), reactant (4 mmol), NaOH (0.4 mmol), solvent: toluene (20 mL), temperature (60 °C), atmosphere (O_2_), and reaction time (6 h).

The stability of catalysts and the reproducibility of the catalyst preparation method are of great importance for heterogeneous catalysis to ensure catalytic performances. The reusability is an important criterion for catalyst evaluation, particularly for practical applications. The reusability test results of the typical 1wt%Au_pre_/Ti_3_Al_x_C_2_T_y_-Air600 are shown in Figure 5a; we found that the catalyst activity and selectivity were almost unchanged in five cycles, which demonstrated the robust catalyst stability, and its potential reasons were that the morphology, composition, and electronic structure remained almost unchanged.

Though the test results confirmed the robust stability in five cycles, it is still necessary to confirm no leaching of active species from 1wt%Au_pre_/Ti_3_Al_x_C_2_T_y_-Air600, namely, no Au NP or TiO_2_ species was detached or dissolved into the reaction solution during the reactions. To this end, we designed and conducted a thermal filtration experiment based on the literature [57,58], and the regular reaction proceeded with solid catalysts for 2 h at the first stage, and then the catalyst was filtered out from the reaction solution. The filtrate with same reaction mixture continued until 6h under identical conditions without solid catalysts. As shown in Figure 5b the catalyst removal stopped the conversion of benzyl alcohol and lost the selectivity of benzaldehyde owing to the consumption caused by unknown homogeneous relations [57,59]. These results could prove that there was no leaching of active species as to the selective oxidation of benzyl alcohol.

### 3.7. Synergistic Effect and Mechanistic Discussions

In the selection of benzyl alcohol over supported metal catalysts, the synergistic effect between metal active centers and support active/adsorptive species was observed to improve catalytic activity [60]. For the catalysts calcined in air in this study, the XRD and TEM analyses identified that TiO_2_ species formed on the catalyst surfaces when calcined in air at 600 °C, and the catalyst of 1wt%Au_pre_/Ti_3_Al_x_C_2_T_y_-Air600 showed improved catalytic performances compared with the catalyst of 1wt%Au_pre_/Ti_3_Al_x_C_2_T_y_. As TiO_2_ is a well-known catalyst for the oxidation of benzyl alcohol, this inspired us to explore whether there is a synergistic effect between the Au NP and TiO_2_ species [61]. To validate this hypothesis, a calculated amount of 1wt%Au_pre_/Ti_3_Al_x_C_2_T_y_ and TiO_2_ was mechanically mixed, and the mass ratio was estimated based on the peak height of anatase TiO_2_ and the support of Ti_3_Al_x_C_2_T_y_ as determined by XRD. The catalytic activity of dual catalysts (physical mixture of anatase TiO_2_ and 1wt%Au_pre_/Ti_3_Al_x_C_2_T_y_) was significantly lower than that of 1wt%Au_pre_/Ti_3_Al_x_C_2_T_y_-Air600, while the selectivity toward benzaldehyde was similar (Figure S10). These results demonstrate that the existence of a synergistic intimate interaction between surface TiO_2_ species and Au NPs to promote the reaction by facilitating the activation of O_2_ or benzyl alcohol.

On the basis of the above findings and the literature reports [62,63], the mechanism was discussed over 1wt%Au_pre_/Ti_3_Al_x_C_2_T_y_-Air600 (Figure S11). Firstly, the O_2_ molecules and benzyl alcohol molecules were activated by active species, and both the TiO_2_ species and Au NPs contributed to this activation, but it was difficult to exactly quantify their individual contribution. Generally, a catalytic process involves adsorption, reaction, and desorption steps. Firstly, a hydrogen bond formed between the hydroxyl groups (-OH) of benzyl alcohol and the support surface of the hydroxyl groups (-OH), and this chemical adsorption helped collect reactants [27]; then, the small amount of base (NaOH) further activated the hydroxyl group (-OH) in benzyl alcohol to generate an alcoholic intermediate [64]. Meanwhile, the adsorbed O_2_ molecules were activated by electrons from the catalysts and formed oxidative species of·O_2_^-^ [65]. Finally, the·O_2_^-^ captured H atoms from the aforementioned alcoholic intermediates to give benzaldehyde and H_2_O [66].

## 4. Conclusions

This work developed and systematically investigated the fluorine-free etching of Ti_3_AlC_2_ MAX phase under basic hydrothermal conditions, which simultaneously reduced HAuCl_4_ to metallic gold by as-released H_2_. By this novel reduction method, a series of Au_pre_/Ti_3_Al_x_C_2_T_y_ catalysts were obtained and exhibited better catalytic performances than those of catalysts prepared by traditional colloidal deposition, chemical reduction, and wet impregnation under identical reaction conditions. Moreover, the effects of calcination temperatures and atmospheres (air, Ar, and H_2_) were also investigated, and the results demonstrate that the catalysts treated by calcination in air outperformed other counterparts treated by Ar or H_2_. In particular, the 1wt%Au_pre_/Ti_3_Al_x_C_2_T_y_-Air600 catalyst exhibited optimistic catalytic performances, probably owing to the formation of tiny surface anatase TiO_2_ species, which had a synergistic effect with Au NPs to promote the reaction rates. The tests of reusability and hot filtration confirmed the stability of the newly developed catalysts. To be frank, this method still has limitations at its current stage as the Au NPs have a large particle size and need optimization work; nonetheless, we believe that this work provides new insights for peer researchers and engineers as to rational catalyst design and preparation, and this method may be applied to other MAX phases or metal precursors.

## Figures and Tables

**Table 1:** Catalytic performances of different catalysts and control experiments.

Catalysts	ICP/wt%	Conversion/%	Selectivity/%	Yield/%	Reaction Rate/mmol·g_Au_^−1^·h^−1^
No catalysts	null	0.8	58.3	0.5	null
Ti_3_AlC_2_	null	0.6	63.0	0.4	null
Ti_3_Al_x_C_2_T_y_	null	3.6	42.5	1.5	null
0.25wt%Au_pre_/Ti_3_Al_x_C_2_T_y_	0.17	4.5	61.4	2.8	54.9
0.5wt%Au_pre_/Ti_3_Al_x_C_2_T_y_	0.32	12.1	68.8	8.3	86.5
1wt%Au_pre_/Ti_3_Al_x_C_2_T_y_	0.67	25.2	88.2	22.2	110.4
1.5wt%Au_pre_/Ti_3_Al_x_C_2_T_y_	1.06	32.8	86.8	28.5	89.6
2wt%Au_pre_/Ti_3_Al_x_C_2_T_y_	1.32	36.0	90.2	32.5	82.1
1wt%Au_pre_/Ti_3_Al_x_C_2_T_y_-Air200	0.73	19.5	99.0	19.5	89.0
1wt%Au_pre_/Ti_3_Al_x_C_2_T_y_-Air400	0.67	23.9	99.0	23.9	118.9
1wt%Au_pre_/Ti_3_Al_x_C_2_T_y_-Air600	0.52	38.5	94.7	36.5	234.0
1wt%Au_pre_/Ti_3_Al_x_C_2_T_y_-Ar200	0.41	14.2	99.0	14.2	115.4
1wt%Au_pre/_Ti_3_Al_x_C_2_T_y_-Ar400	0.76	5.0	99.0	5.0	21.9
1wt%Au_pre_/Ti_3_Al_x_C_2_T_y_-Ar600	0.68	1.6	99.0	1.6	7.8
1wt%Au_pre_/Ti_3_Al_x_C_2_T_y_-H200	0.67	12.2	89.8	11.0	60.7
1wt%Au_pre/_Ti_3_Al_x_C_2_T_y_-H400	0.55	10.4	91.1	9.5	57.6
1wt%Au_pre_/Ti_3_Al_x_C_2_T_y_-H600	0.71	1.6	52.6	0.8	3.8

Reaction conditions: Catalyst (200 mg), reactant (4 mmol), NaOH (0.4 mmol), solvent: toluene (20 mL), temperature (60 °C), atmosphere (O_2_), and reaction time (6 h).

**Table 2:** Comparison of catalytic performances of different gold loading methods.

Catalysts	Conversion/%	Selectivity/%	Yield/%
Au_pre_/Ti_3_Al_x_C_2_T_y_	25.2	88.2	22.2
Au_col_/Ti_3_Al_x_C_2_T_y_	16.6	96.3	16.0
Au_cr_/Ti_3_Al_x_C_2_T_y_	7.7	94.1	7.3
Au_wi_/Ti_3_Al_x_C_2_T_y_	10.2	93.1	9.5
Au_pre_/Ti_3_Al_x_C_2_T_y_-Air600	38.5	94.7	36.5
Au_col_/Ti_3_Al_x_C_2_T_y_-Air600	30.2	88.4	26.7
Au_cr_/Ti_3_Al_x_C_2_T_y_-Air600	9.1	99.9	9.1
Au_wi_/Ti_3_Al_x_C_2_T_y_-Air600	8.6	89.7	7.7

Reaction conditions: Catalyst (200 mg), reactant (4 mmol), NaOH (0.4 mmol), solvent: toluene (20 mL), temperature (60 °C), atmosphere (O_2_), and reaction time (6 h).

**Table 3:** Comparison of aromatic alcohols over 1wt%Au_pre_/Ti_3_Al_x_C_2_T_y_-Air600 catalysts.

Reagent	Target Product	Conversion/%	Selectivity/%	Yield/%	Reaction Rate/mol·g_Au_^−1^·h^−1^
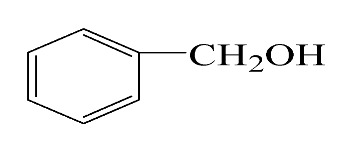	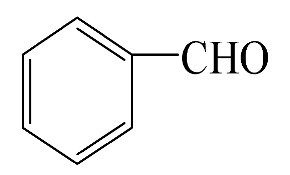	38.5	94.7	36.5	234.0
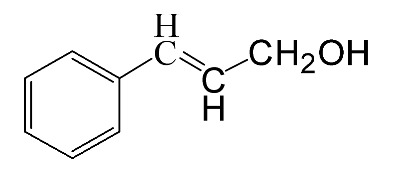	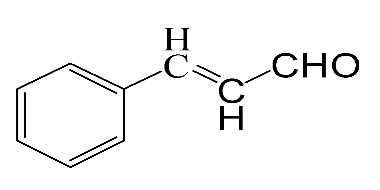	3.4	>99	3.4	21.8
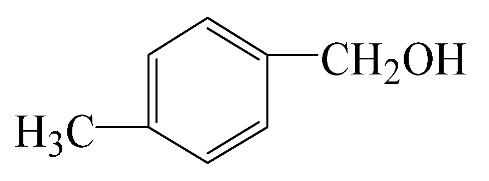	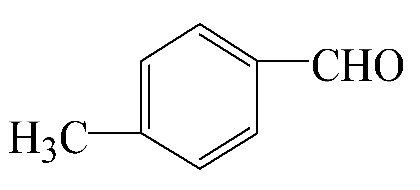	49.3	88.0	43.3	277.6
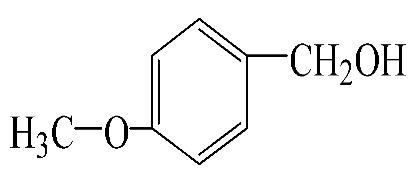	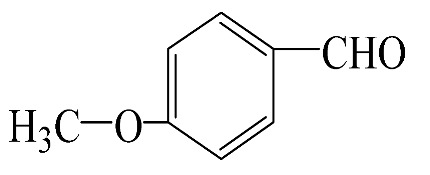	55.3	96.5	53.4	342.3

Reaction conditions: Catalyst (200 mg), reactant (4 mmol), NaOH (0.4 mmol), solvent: toluene (20 mL), temperature (60 °C), atmosphere (O_2_), and reaction time (6 h).

## Supplementary Materials

The following supporting information can be downloaded at: https://www.mdpi.com/article/10.3390/ma16083139/s1, Figure S1: XRD patterns of (a) original Ti_3_AlC_2_; (b) 0.25wt%Au/Ti_3_Al_x_C_2_T_y_;(c) 0.5wt%Au/Ti_3_Al_x_C_2_T_y_; (d) 1wt%Au/Ti_3_Al_x_C_2_T_y_; (e) 1.5wt%Au/Ti_3_Al_x_C_2_T_y_; and (f) 2wt%Au/Ti_3_Al_x_C_2_T_y_. Figure S2: XRD patterns of Ti_3_Al_x_C_2_T_y_ support and the calcined samples in air: Ti_3_Al_x_C_2_T_y_-Air200, Ti_3_Al_x_C_2_T_y_-Air400, and Ti_3_Al_x_C_2_T_y_-Air600. Figure S3: High-resolution XPS analyses of Au4f over 1wt%Au_cr_/Ti_3_Al_x_C_2_T_y_. Table S1: Summarized binding energies of the metallic gold (Au), the positively-charged gold (Au^δ+^) and the negatively-charged gold (Au^δ-^) obtained from literature reports. Table S2: Detailed binding energies and fractions of sub-species deconvoluted from high-resolution XPS spectra of C1s and O1s. Figure S4: TEM images of (a) 1wt%Au/Ti_3_Al_x_C_2_T_y_-Air200; (b) 1wt%Au/Ti_3_Al_x_C_2_T_y_-Air400. Figure S5: SEM images of (a) 1wt%Au_pre_/Ti_3_Al_x_C_2_T_y_; (b) 1wt%Au_pre_/Ti_3_Al_x_C_2_T_y_-Air600; (c) 1wt%Au_pre_/Ti_3_Al_x_C_2_T_y_-Ar600; (d) 1wt%Au_pre_/Ti_3_Al_x_C_2_T_y_-H600. Figure S6: Effect of different catalyst dosages using 1wt%Au_pre_/Ti_3_Al_x_C_2_T_y_-Air600. Figure S7: Effect of atmospheres using 1wt%Au_pre_/Ti_3_Al_x_C_2_T_y_-Air600. Figure S8: Effect of different reaction temperatures using 1wt%Au_pre_/Ti_3_Al_x_C_2_T_y_-Air600. Figure S9: The conversion of benzyl alcohol and selectivity of benzaldehyde over series of 1wt%Au/Ti_3_Al_x_C_2_T_y_-Air catalysts (a,b), 1wt%Au/Ti_3_Al_x_C_2_T_y_-Ar (c, d), and 1wt%Au/Ti_3_Al_x_C_2_T_y_-H (e, f). Figure S10: Synergistic effect between surface TiO_2_ species and Au NPs. Table S3: Summary of selective oxidation of benzyl alcohol to benzaldehyde in liquid phase system by supported gold catalyst. Figure S11: The schematic illustration of catalyst preparation process and reaction mechanism for the selective oxidation of benzyl alcohol to benzaldehyde.
