# Supplementary material for: Systematic Investigation on Supported Gold Catalysts Prepared by Fluorine-Free Basic Etching Ti3AlC2 in Selective Oxidation of Aromatic Alcohols to Aldehydes
Source: Materials (Basel). 2023 Apr 16;16(8):3139. doi: 10.3390/ma16083139 (PMC10143880; doi:10.3390/ma16083139)
Supplement: Supplementary file 1 [file materials-16-03139-s001.zip › materials-2322462-supplementary.pdf]

Article

Systematic Investigation on Supported Gold Catalysts Prepared by Fluorine-Free Basic Etching Ti_3AlC_2 in Selective Oxidation of Aromatic Alcohols to Aldehydes

Hangwei Jiang, Xiya Chen, Danlan Cui, Kun Lu and Xiao Kong and Xingguang Zhang *

School of Materials and Chemistry, University of Shanghai for Science and Technology, 516 Jungong Road, Shanghai 200093, China

* Correspondence: x.g.zhang@usst.edu.cn

Section 1

XRD patterns of the original Ti_3AlC_2 and enlarged XRD patterns of series of $Au_{pre}/Ti_3Al_xC_2T_y$ catalysts to identify the characteristic peaks of Au NPs: Au(111), Au(200), and Au(220).

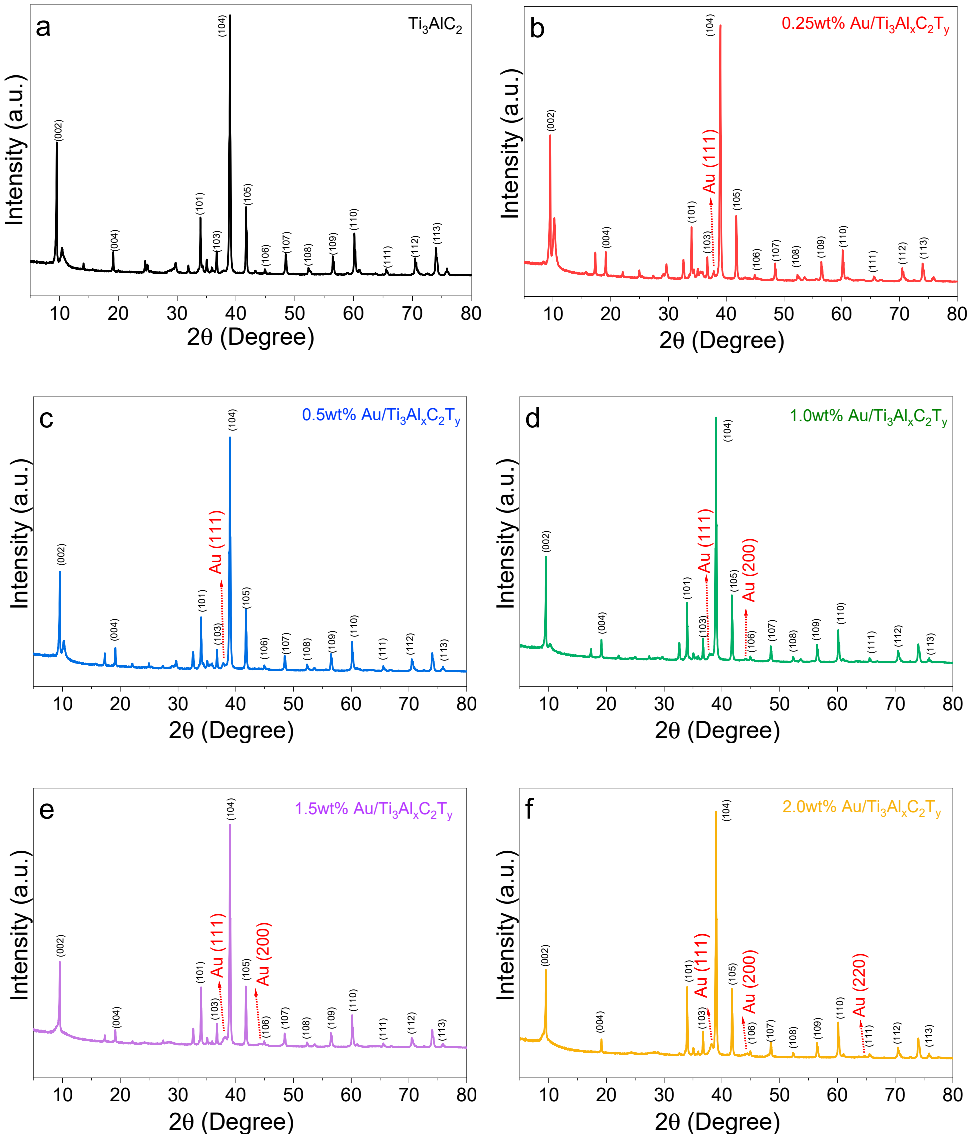

Figure S1. XRD patterns of **(a)** original Ti_3AlC_2; **(b)** 0.25wt%Au/$Ti_3Al_xC_2T_y$;**(c)** 0.5wt%Au/$Ti_3Al_xC_2T_y$; **(d)** 1wt%Au/$Ti_3Al_xC_2T_y$; **(e)** 1.5wt%Au/$Ti_3Al_xC_2T_y$; and **(f)** 2wt%Au/$Ti_3Al_xC_2T_y$.

Section 2

XRD patterns of the $Ti_3Al_xC_2T_y$ support and its calcined samples in air at 200ºC, 400ºC and 600ºC. To confirm the formation of surface TiO_2 species on $Ti_3Al_xC_2T_y$-Air600.

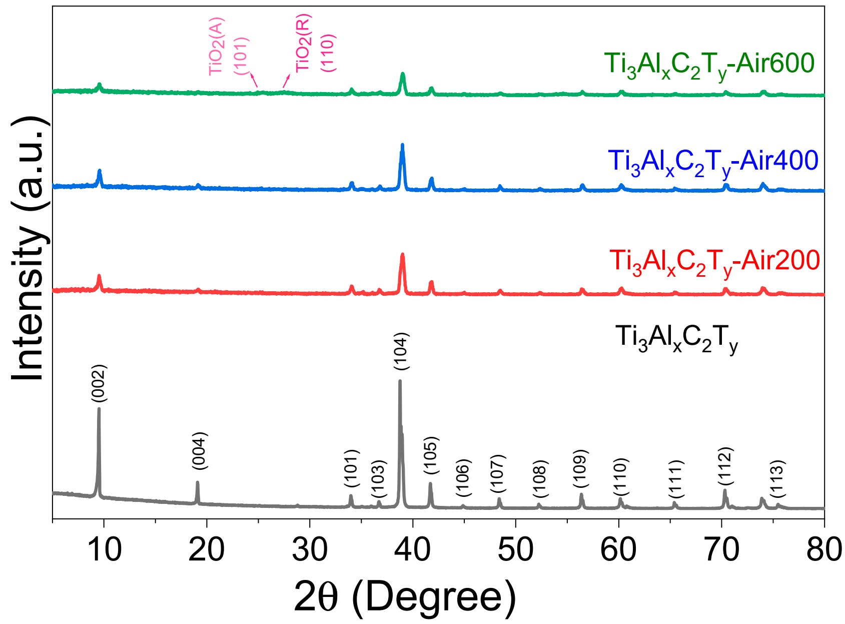

Figure S2. XRD patterns of $Ti_3Al_xC_2T_y$ support and the calcined samples in air: $Ti_3Al_xC_2T_y$-Air200, $Ti_3Al_xC_2T_y$-Air400, and $Ti_3Al_xC_2T_y$-Air600.

Section 3

XPS analysis of control catalyst (1wt%Au_{cr}/$Ti_3Al_xC_2T_y$) prepared by traditional chemical reduction method.

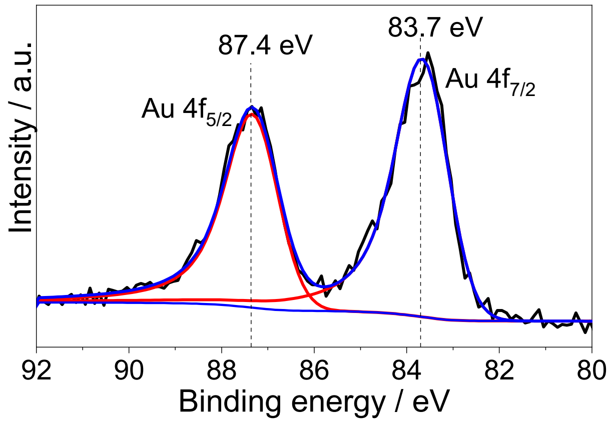

Figure S3. High-resolution XPS analyses of Au4f over 1wt%Au_{cr}/$Ti_3Al_xC_2T_y$.

Section 4

XPS analysis results of Au4f, C1s, O1s, and Ti2p from literature and the quantified proportions of formed species associated with C1s, O1s and Ti2p.

Table S1. Summarized binding energies of the metallic gold (Au), the positively-charged gold ($Au^{\delta+}$) and the negatively-charged gold ($Au^{\delta-}$) obtained from literature reports.

Material	$Au^{\delta+}$ ($4f_{7/2}$) / eV	Au ($4f_{7/2}$) /eV	$Au^{\delta-}$ ($4f_{7/2}$) /eV	Ref
$Au_{pre}/Ti_3Al_xC_2T_y$		83.5	--	This work
$Au_{cr}/Ti_3Al_xC_2T_y$		83.7	--	This work
$Au@TiO_{2-x}/ZnO$	-	83.5	83.1	[s1]
$TiO_x@Au/TiO_2$	-	83.5	83.1	[s2]
$Au@Ti/SiO_2$-wcSMSI	-	83.8	83.4	[s3]
Au-BiOCl	-	83.4	-	[s4]
Au/TiC	-	84	-	[s5]
Au/TiO_2-800	-	83.2	-	[s5]
Au/HAP-800	-	83.8	-	[s6]
Au/CeO_2	85.5 (Au^+)	83.8	-	[s7]
Au/CeO_2	85.9 (Au^+)	84	-	[s8]
$Au-CeO_2$@SBA-15	84.6 (Au^+)<br>86 (Au^{3+})	84	-	[s9]
Au/FeO_x-TiO_2	86.3 (Au^{3+})	84	-	[s10]

Note:—refers to unavailable or undetectable.

Table S2. Detailed binding energies and fractions of sub-species deconvoluted from high-resolution XPS spectra of C1s and O1s.

Items		**$Au_{pre}/Ti_3Al_xC_2T_y$**		**$Au_{pre}/Ti_3Al_xC_2T_y$-Air600**		**$Au_{pre}/Ti_3Al_xC_2T_y$-Ar600**		**$Au_{pre}/Ti_3Al_xC_2T_y$-H600**	
		B.E. / eV	Fraction / %	B.E. / eV	Fraction / %	B.E. / eV	Fraction / %	B.E. / eV	Fraction / %
**C1s**	**Ti-C**	281.9	9.5	281.9	0.5	281.6	2.4	281.6	3.1
	**C-C**	284.8	63.1	284.8	72.8	284.8	74.8	284.8	73.6
	**C-O**	286.5	16.8	286.3	18.1	286.4	14.1	286.4	13.7
	**O-C=O**	288.8	10.7	288.8	8.6	288.9	8.8	288.9	9.6
**O1s**	**Ti-O/Ti=O**	529.4	77.6	529.2	74.2	529.2	59.7	528.9	65.6
	**O vacancy**	530.9	12.9	530.9	12.5	530.8	20.0	530.6	21.4
	**O-H/Na**	532.3	6.4	532.5	7.3	532.0	13.9	532.0	9.5
	**$O_{adsorbed}$**	534.6	3.2	534.5	2.0	533.3	6.4	534.1	3.6
**Ti2p**	**Ti^{3+}/Ti^{4+}**	0.23		Undetectable		0.12		0.21	

Section 5

Particle size distribution analysis by TEM analysis and strong metal-support interaction (SMSI) observation by HR-TEM analysis.

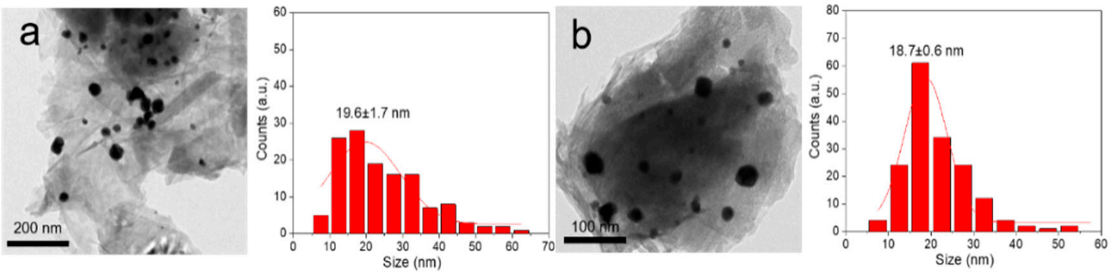

Figure S4. TEM images of **(a)** 1wt%Au/$Ti_3Al_xC_2T_y$-Air200; **(b)** 1wt%Au/$Ti_3Al_xC_2T_y$-Air400.

Section 6

SEM images of four typical catalysts

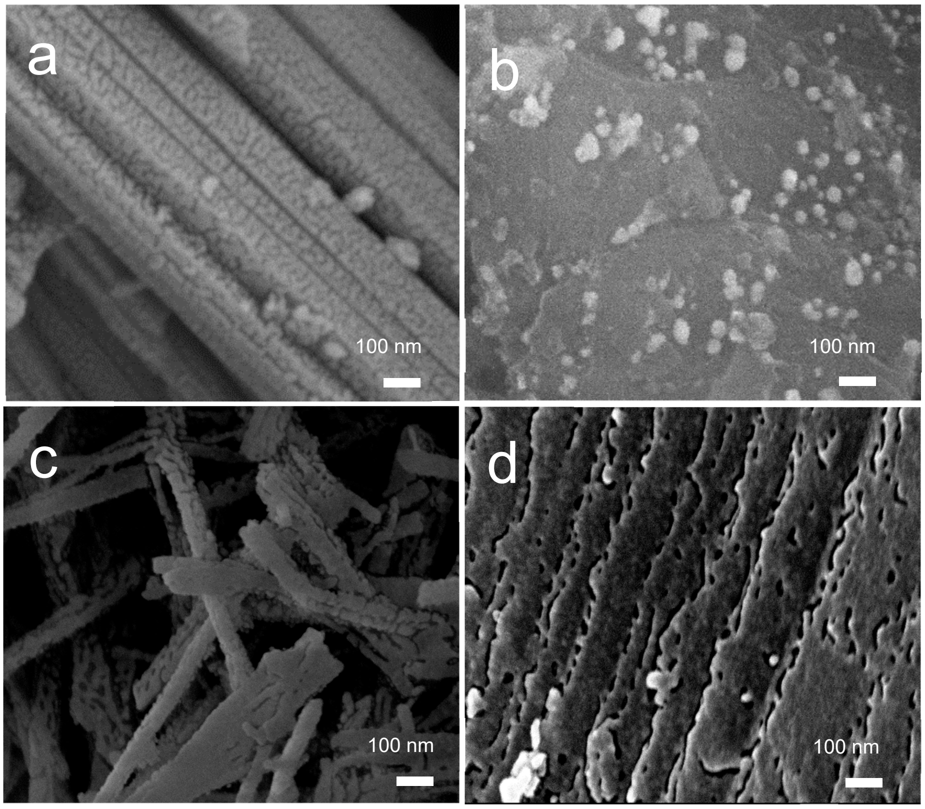

Figure S5. SEM images of **(a)** 1wt%Au_{pre}/$Ti_3Al_xC_2T_y$; **(b)** 1wt%Au_{pre}/$Ti_3Al_xC_2T_y$-Air600; **(c)** 1wt%Au_{pre}/$Ti_3Al_xC_2T_y$-Ar600; **(d)** 1wt%Au_{pre}/$Ti_3Al_xC_2T_y$-H600.

Section 7

Optimization work on catalyst mass, reaction temperatures and comparison of reaction atmospheres (O_2, air or N_2) and reaction time.

Catalyst dosage: The effect of different catalyst dosages was conducted using 1wt%Au_{pre}/$Ti_3Al_xC_2T_y$-Air600 and the results were given in **Figure s6**. It showed that increasing the catalyst dosage significantly enhanced the conversion of benzyl alcohol. In particular, the conversion of benzyl alcohol increased from 25% to 73% when the amount of catalyst increased from 0.1g to 0.2g, whereas the conversion of benzyl alcohol improved little when the catalyst mass increased from 0.2g to 0.3g, showing that 0.2g in this reaction system was the optimum amount.

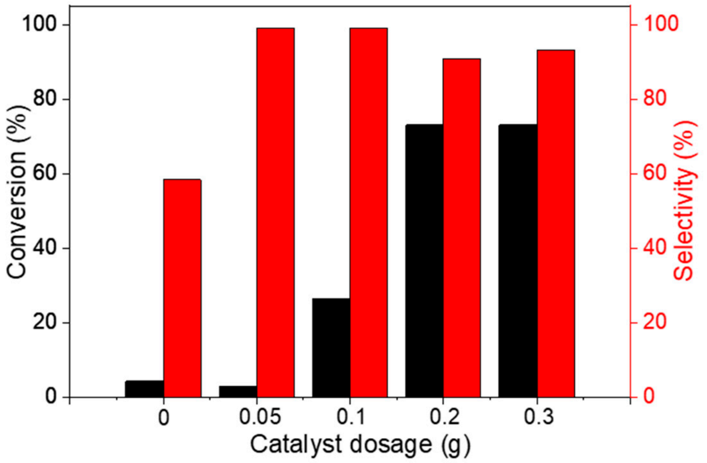

Figure S6. Effect of different catalyst dosages using 1wt%Au_{pre}/$Ti_3Al_xC_2T_y$-Air600. **Reaction conditions**: catalyst mass (variant), benzyl alcohol (4 mmol), sodium hydroxide (0.4 mmol), solvent of toluene (20 ml), reaction temperature (90°C), atomosphere (O_2), reaction time (6 h).

Effect of atmosphere: Since O_2 plays a unique role in the selective oxidation of benzyl alcohol to benzaldehyde, in order to confirm the role of O_2, we conducted three sets of experiments using 1wt%Au_{pre}/$Ti_3Al_xC_2T_y$-Air600 in the atmosphere of N_2, Air and O_2, respectively. As shown in **Figure S7**, the selective oxidation of benzyl alcohol over 1wt%Au_{pre}/$Ti_3Al_xC_2T_y$-Air600 greatly depended on the content of O_2, in which the conversion of benzyl alcohol reached 73% in O_2 atmosphere. In the atmosphere of N_2 and air, the conversion of benzyl alcohol was only 0.6% and 38.5%. It showed that O_2 played an important role in the oxidation of benzyl alcohol.

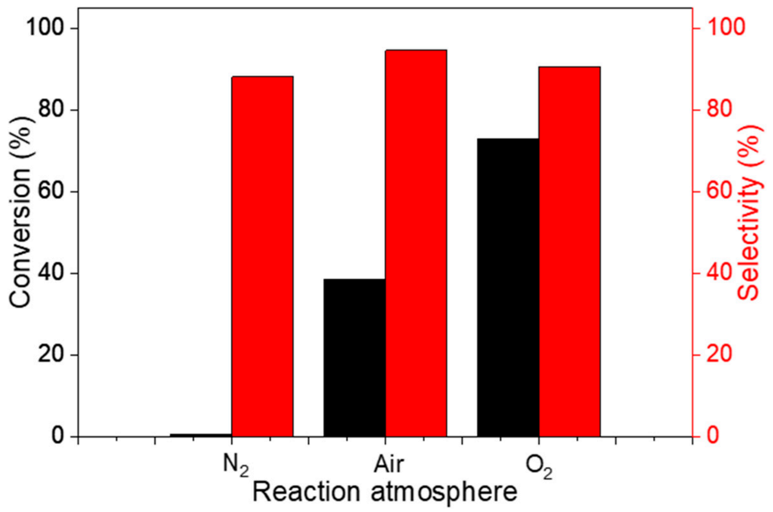

Figure S7. Effect of atmospheres using 1wt%Au_{pre}/$Ti_3Al_xC_2T_y$-Air600. **Reaction conditions**: catalyst mass (200 mg), benzyl alcohol (4 mmol), sodium hydroxide (0.4 mmol), solvent of toluene (20 ml), reaction temperature: (90°C), atmosphere (variant), reaction time (6 h).

Effect of temperatures: we also studied the thermal effect on the selective oxidation of benzyl alcohol catalyzed over 1wt%Au_{pre}/$Ti_3Al_xC_2T_y$-Air600. As shown in **Figure S8**, at room temperature (25 °C) and 60 °C, the conversion of benzyl alcohol was 0.9% and 38.5%, respectively, which was much lower than the 73% conversion at 90 °C. It showed that the reaction temperature also plays an important role in the oxidation of benzyl alcohol. Considering the volatile properties of toluene, 60°C was selected as the typical reaction in this study.

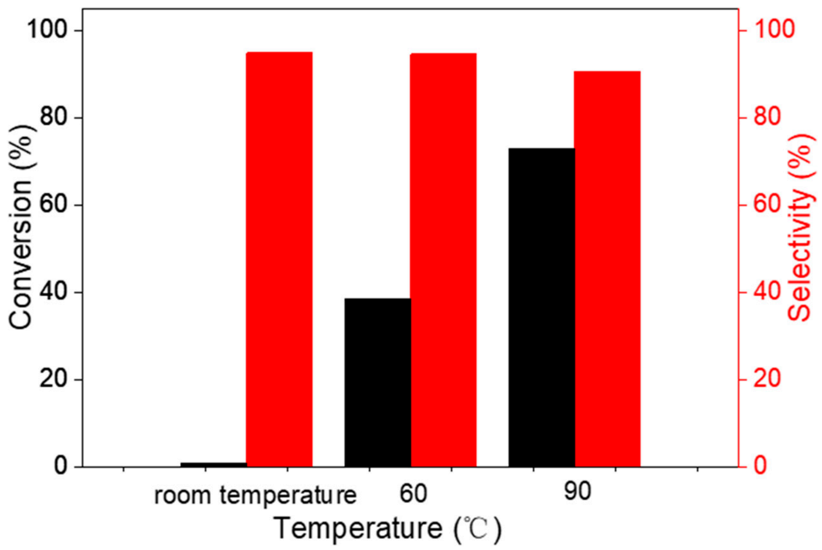

Figure S8. Effect of different reaction temperatures using 1wt%Au_{pre}/$Ti_3Al_xC_2T_y$-Air600. **Reaction conditions**: catalyst mass (200 mg), benzyl alcohol (4 mmol), sodium hydroxide (0.4 mmol), solvent of toluene (20 ml), reaction temperature: (variant), atmosphere (O_2), reaction time (6 h).

Effect of reaction time: Results in **Table 1** only showed the catalytic performances at 6h. To further understand the reaction as the function of time. **Figure S9** showed the conversion and selectivity of series of catalysts calcined in air, Ar and H_2, respectively, and exhibiting that the catalytic conversions went up as the reaction time increased. The air-calcined samples outperformed their counterparts treated by Ar or H_2.

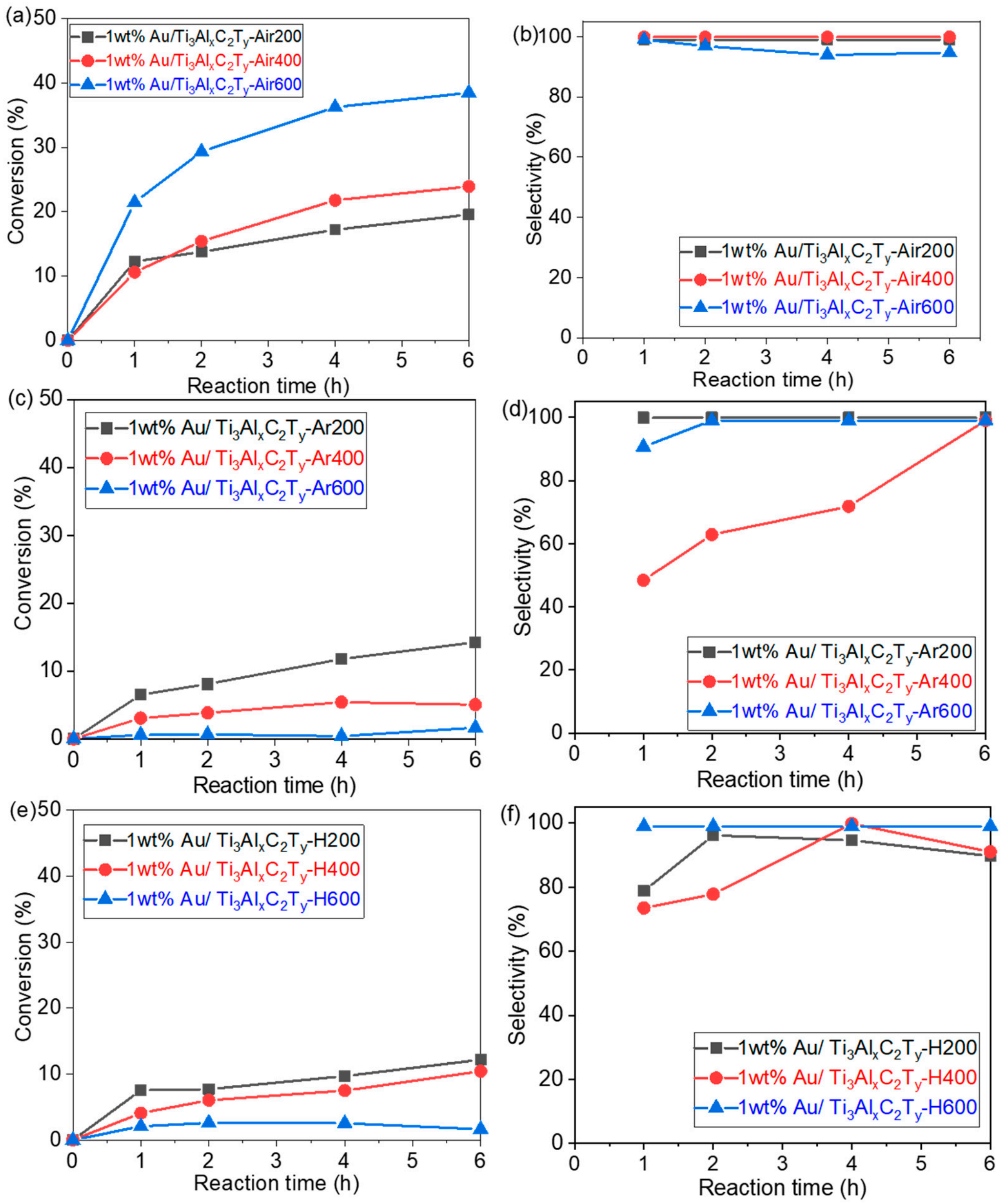

Figure S9. The conversion of benzyl alcohol and selectivity of benzaldehyde over series of 1wt%Au/$Ti_3Al_xC_2T_y$-Air catalysts **(a, b),** 1wt%Au/$Ti_3Al_xC_2T_y$-Ar **(c, d)**, and 1wt%Au/$Ti_3Al_xC_2T_y$-H **(e, f)**. **Reaction conditions**: Catalyst (200 mg), reactant (4 mmol), sodium hydroxide (0.4 mmol), solvent: toluene (20 mL), temperature (60°C), atmosphere (O_2), reaction time (variant).

Section 8

Confirmation of synergistic effect between surface TiO_2 species and Au NPs

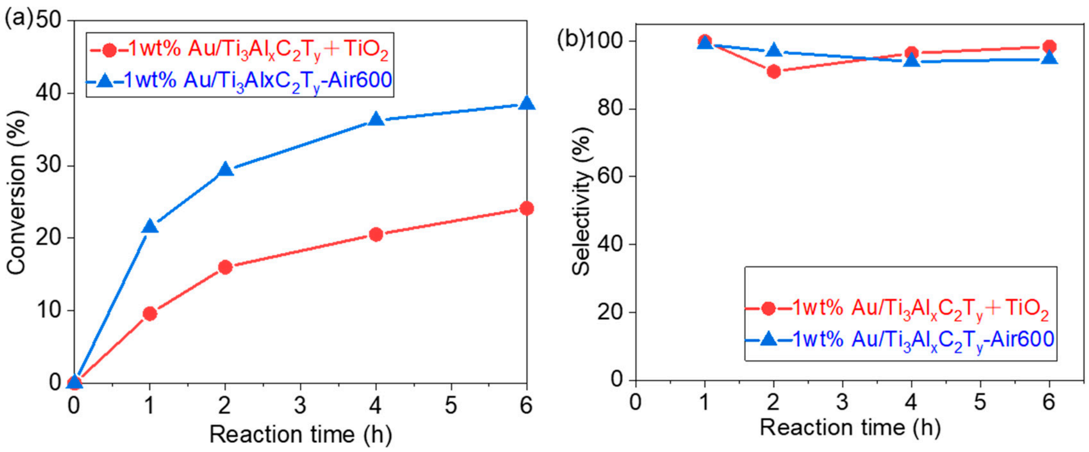

Figure S10. Synergistic effect between surface TiO_2 species and Au NPs. (a) The conversion of benzyl alcohol over 1wt%Au/$Ti_3Al_xC_2T_y$-Air600 and 1wt%Au/$Ti_3Al_xC_2T_y$+TiO_2 catalysts, (b) The selectivity of benzaldehyde over 1wt%Au/$Ti_3Al_xC_2T_y$-Air600 and 1wt%Au/$Ti_3Al_xC_2T_y$+TiO_2 catalysts. **Reaction conditions**: Catalyst (200 mg), reactant (4 mmol), sodium hydroxide (0.4 mmol), solvent: toluene (20 mL), temperature (60°C), atmosphere (O_2), reaction time (6h).

Section 9

Table S3. Summary of selective oxidation of benzyl alcohol to benzaldehyde in liquid phase system by supported gold catalyst.

Catalysts	Oxidant	Reaction time	Reaction temperature	Conversion / %	Selectivity / %	Yield / %	References
$Au_{pre}/Ti_3Al_xC_2T_y$	O_2	6h	60℃	25.2	88.2	22.2	This work
$Au_{pre}/Ti_3Al_xC_2T_y$-Air600	O_2	6h	60℃	38.5	94.7	36.5	This work
Au/SiO_2	O_2	3h	100℃	56	29	16.2	[s11]
Au/TiO_2	O_2	3h	100℃	63	32	20.2	[s11]
Au/Al_2O_3	O_2	3h	100℃	76	22	16.7	[s11]
Au/UiO-66	O_2	10h	80℃	53.8	53.7	28.9	[s12]
Au/NPC	O_2	8h	60℃	98.9	>99	98.9	[s13]
Au/PC	O_2	8h	60℃	51.4	>99	51.4	[s13]

Section 11

Illustration of the catalyst preparation process and reaction mechanism.

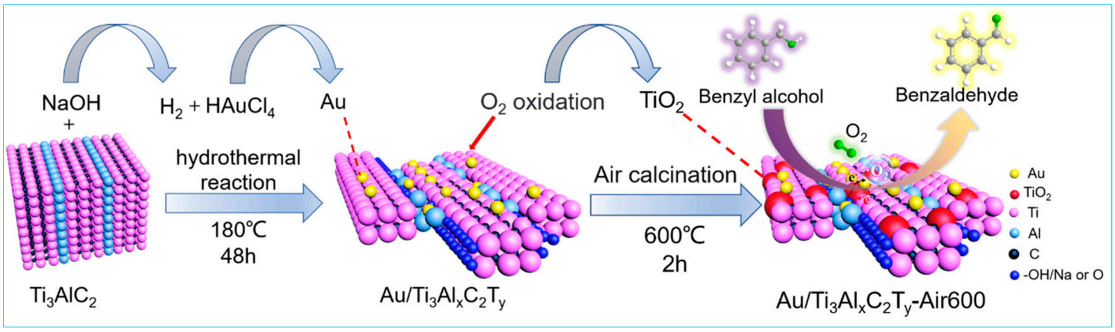

Figure S11. The schematic illustration of catalyst preparation process and reaction mechanism for the selective oxidation of benzyl alcohol to benzaldehyde.

References

[s1] N. Liu, M. Xu, Y. S. Yang, S. M. Zhang, J. Zhang, W. L. Wang, L. R. Zheng, S. Hong, M. Wei, *$Au^{\delta-}$-O_v-Ti^{3+} interfacial site: catalytic active center toward low-temperature water gas shift reaction*, **ACS Catal.**, 2019, 9, 2707-2717.

[s2] H. L. Tang, Y. Su, B. S. Zhang, A. F. Lee, M. A. Isaacs, K. Wilson, L. Li, Y. G. Ren, J. H. Huang, M. Haruta, B. T. Qiao, X. Liu, C. Z. Jin, D. S. Su, J. H. Wang, T. Zhang, *Classical strong metal-support interactions between gold nanoparticles and titanium dioxide*, **Sci. Adv.**, 2017, 3, 1700231.

[s3] J. Zhang, H. Wang, L. Wang, S. Ali, C. T. Wang, L. X. Wang, X. J. Meng, B. Li, D. S. Su, F. S. Xiao, *Wet-chemistry strong metal-support interactions in titania-supported Au catalysts*, **J. Am. Chem. Soc.**, 2019, 141, 2975-2983.

[s4] H. Li, F. Qin, Z. P. Yang, X. M. Cui, J. F. Wang, L. Z. Zhang, *New reaction pathway induced by plasmon for selective benzyl alcohol oxidation on BiOCl possessing oxygen vacancies*, **J. Am. Chem. Soc.**, 2017, 139, 3513-3521.

[s5] J. H. Kim, H. Woo, J. W. Choi, H. W. Jung, Y. T. Kim, *CO_2 electroreduction on Au/TiC: enhanced activity due to metal-support interaction*, **ACS Catal.**, 2017, 7, 2101-2106.

[s6] H. L. Tang, F. Liu, J. K. Wei, B. T. Qiao, K. F. Zhao, Y. Su, C. Z. Jin, L. Li, J. Y. Liu, J. H. Wang, T. Zhang, *Ultrastable hydroxyapatite /titanium dioxide supported gold nanocatalyst with strong metal-support interaction for carbon monoxide oxidation*, **Angew Chem. Int. Edit.**, 2016, 55, 10606-10611.

[s7] X. S. Huang, H. Sun, L. C. Wang, Y. M. Liu, K. N. Fan, Y. Cao, *Morphology effects of nanoscale ceria on the activity of Au/CeO_2 catalysts for low-temperature CO oxidation*, **Appl. Catal. B.**, 2009, 90, 224-232.

[s8] R. Leppelt, B. Schumacher, V. Plzak, M. Kinne, R. J. Behm, *Kinetics and mechanism of the low-temperature water-gas shift reaction on Au/CeO_2 catalysts in an idealized reaction atmosphere*, **J. Catal.**, 2006, 244, 137-152.

[s9] T. Wang, X. Yuan, S. R. Li, L. Zeng, J. L. Gong, *CeO_2-modified Au@SBA-15 nanocatalysts for liquid-phase selective oxidation of benzyl alcohol*, **Nanoscale**, 2015, 7, 7593-7602.

[s10] Y. F. Yang, P. Sangeetha, Y. W. Chen, *Au/FeO_x-TiO_2 catalysts for the preferential oxidation of CO in a H_2 stream*, **Ind. Eng. Chem. Res.**, 2009, 48, 10402-10407.

[s11] J. A. D. Gualteros, M. A. S. Garcia, A. G. M. da Silva, T. S. Rodrigues, E. G. Cândido, F. A. e Silva, F. C. Fonseca, J. Quiroz, D. C. de Oliveira, S. I. C. de Torresi, C. V. R. de Moura, P. H. C. Camargo, E. M. de Moura, *Synthesis of highly dispersed gold nanoparticles on Al_2O_3, SiO_2, and TiO_2 for the solvent-free oxidation of benzyl alcohol under low metal loadings*, **Journal of Materials Science.**, 2019, 54, 238-251.

[s12] J. Zhu, P. C. Wang, M. Lu, *Selective oxidation of benzyl alcohol under solvent-free condition with gold nanoparticles encapsulated in metal-organic framework*, **Applied Catalysis A: General.**, 2014, 477, 125-131.

[s13] D. Mao, M. Jia, J. Qiu, X. F. Zhang, J. Yao, *N-doped porous carbon supported Au nanoparticles for benzyl alcohol oxidation*, **Catalysis Letters.**, 2020, 150, 74-81.
